# Therapeutic Potential of Irisin in Neurodegenerative Diseases

**DOI:** 10.3390/ijms262311348

**Published:** 2025-11-24

**Authors:** Sania Muzaffar, Alpna Tyagi, Subbiah Pugazhenthi

**Affiliations:** 1 Rocky Mountain Regional VA Medical Center, Aurora, CO 80045, USA; 2 Department of Medicine, University of Colorado-Anschutz Medical Campus, Aurora, CO 80045, USA

**Keywords:** irisin, FNDC5, mitochondria, neuroprotection, aging, alzheimer’s disease

## Abstract

Irisin is a myokine secreted by muscle in response to exercise. It is derived from a transmembrane protein called fibronectin type III domain-containing protein 5 (FNDC5). The *Fndc5* gene is expressed in several tissues, including skeletal muscle, brain, adipose tissue, heart, kidney, and lung. Irisin is cleaved from FNDC5 protein by the enzyme furin and released into circulation. In addition to exercise, several drugs have been shown to increase the production of irisin. Administration of exogenous irisin mimics the beneficial actions of exercise. Irisin can cross the blood–brain barrier and exert neuroprotective actions in the brain. It has been shown to reverse Alzheimer’s pathologies in clinical and animal studies. Irisin also exerts protective effects against obesity, diabetes, and cardiovascular disease, diseases that often coexist with aging AD patients. Multiple approaches have been taken to suggest that exercise may act through irisin. Studies have provided direct evidence linking the two using *Fndc5* gene deletion and irisin antibodies. Irisin binds to αVβ1/β5 integrins to mediate the activation of integrin-FAK pathways. While exercise as a lifestyle modification for healthy aging is well recognized, it may present limitations in some aging populations, especially those with disease conditions, including Parkinson’s disease. Administration of exogenous irisin or small molecules that increase the expression of endogenous irisin or facilitate its actions are some alternate approaches that can mimic the beneficial actions of exercise. This review discusses the therapeutic potential of irisin in the treatment of neurodegenerative and other aging-associated diseases.

## 1. Introduction

The technological advancements of the 21st century, including TV, video games, and computers, have led people to spend several hours in a sedentary state at home and in the workplace [1,2,3]. These lifestyles contribute to age-related diseases, such as diabetes, cardiovascular diseases, and neurodegenerative diseases. Moreover, according to the World Health Organization (WHO), physical inactivity is a major risk factor for mortality [4]. It is increasingly recognized that physical activity and exercise have a positive impact on the brain, helping to prevent or slow cognitive decline in patients with dementia [5,6]. Preserving cognitive function is a major challenge during aging, one of the key factors that contributes to cognitive decline. Interestingly, exercise, particularly endurance exercise, is known to improve cognitive function during aging.

The positive effects of exercise in Alzheimer’s disease (AD) are well documented. A study by Morris et al., 2017, reported that 150 min/week of aerobic exercise in early AD (mean age, 72.9) benefits functional ability (Disability Assessment for Dementia), reduces depressive symptoms (Cornell Scale for Depression in Dementia), improves memory performance, and reduces hippocampal atrophy [7]. Voluntary exercise for five months reduces Aβ levels and amyloid deposition in the brain of TgCRND8 transgenic AD mice [8,9]. Long-term exercise enhances the rate of learning in these mice, as shown using the Morris water maze [8]. Treadmill exercise of moderate intensity (60 min/day; 5 days/week) for 4 or 12 weeks reduces Aβ and proinflammatory protein levels in TgAPP/PS1ΔE9 mice [10]. In contrast, sedentary APP/PS1 mice show more Aβ plaques in the hippocampus and reduce “central crossing” in the open field test compared to APP/PS1 mice performing resistance exercise [11]. Short-term resistance training (climbing up a 1 m ladder with a progressively heavier weight loading) improves cognitive function and reduces neuropathological and neuroinflammatory markers in the frontal cortex and hippocampus of nine-month-old 3 × Tg mice [12]. Both high-intensity interval training (HIIT) and moderate-intensity continuous exercise (MICE) interventions improve neurocognitive performance [i.e., reaction times (RTs) and event-related potential (ERP) P3 latency and amplitudes] [13]. Nine months of exercise on running wheels decreases hippocampal Tau pathology, another hallmark of AD in 3-month-old THY-Tau22 mice [14,15,16,17]. Thus, regular physical activity is beneficial in preventing/delaying the onset and progression of AD. In this review, we examine the molecular mechanisms by which exercise exerts positive effects on the brain.

## 2. Irisin Is a Novel Exercise-Induced Molecule

Irisin is a hormone secreted by the skeletal muscle in response to exercise. It is derived from a protein called fibronectin type III domain-containing protein 5 (FNDC5), a glycosylated 209-amino acid transmembrane protein. It consists of a C-terminal hydrophobic transmembrane domain and an N-terminal signal peptide, a fibronectin type III domain [18]. The *Fndc5* gene is predominantly expressed in skeletal muscle, but its expression has been reported in other tissues, including the brain, adipose tissue, heart, kidney, and lung [19]. *Fndc5* expression varies throughout developmental stages, with the highest expression detected in gastrocnemius muscles of mature mice and the lowest in pups [20]. Adult-born neurons in dentate gyrus are morphologically and functionally abnormal in *Fndc5* knockout mice [21]. Moreover, FNDC5 levels are reduced in the postmortem brain tissues of patients with major depressive disorder (MDD) and in mice exhibiting depressive-like behavior [22].

Free wheel running exercise for thirty days has been shown to induce hippocampal *fndc5* gene expression in 6-week-old male wild-type mice [23]. Moreover, free wheel running exercise for 3 weeks followed by 12 h rest in wild-type mice (12-week-old) increases *fndc5* mRNA expression in skeletal muscles [18]. Exercise-regulated *fndc5* expression involves αV/β5 integrin receptor [24], peroxisome proliferator-activated receptor gamma coactivator-1 alpha (PGC-1α) [18], and diverse signaling pathways, including AMP-activated protein kinase (AMPK) and p38 mitogen-activated protein kinase (MAPK) [18,23]. In addition, exercise elevates Ca^2+^ in muscle cytoplasm, which further activates the AMPK-PGC-1α-FNDC5 axis for irisin synthesis [25].

The extracellular region of FNDC5 (the N-terminal fragment) is proteolytically cleaved by furin, a pro-protein convertase enzyme, and secreted into the circulation as irisin, named after Iris, a Greek messenger and rainbow goddess [18]. Irisin is a conserved protein with 100% similarity between mouse and human [26]. Its circulating basal level, determined via Mass Spectrometry, is 3–4 ng/mL in humans [27] and 0.3 ng/mL in mouse plasma [24]. However, several studies have reported a wide range of concentrations for human and mouse irisin levels measured with ELISAs [28]. A major reason for the discrepancy in circulating irisin concentrations is the lack of specificity of irisin antibodies used in commercially available assay kits, resulting in higher reported levels of irisin [29]. Irisin levels decrease with age [30] and in AD patients compared to healthy controls [31]. Therefore, it is essential to understand how irisin secretion is regulated.

## 3. Regulation of Irisin Secretion

Physical exercise has been shown to be a potent inducer of irisin secretion in both humans and rodents [18,32]. Different types of exercise have been tested for irisin response. Resistance training styles, including chest press, lat pull down, leg press, knee extension, seated rowing, shoulder press, arm curl, and tricep press down, induce irisin in healthy young adults in their twenties [33,34]. Ten weeks of endurance exercise (aerobic) leads to a two-fold increase in plasma irisin levels in healthy adult male subjects compared to those in a non-exercised state [18]. Exercise-induced irisin secretion has been observed in the elderly population as well. For example, Zhao et al. (2017) reported that 12 weeks of resistance exercise induces approximately a two-fold increase in plasma levels of irisin in old male adults (mean age of 62.1 years) [35]. In obese females, single bouts of afternoon isocaloric exercise of different intensities (moderate vs. high intensity) show an increase in plasma irisin levels compared to baseline [36]. Aerobic exercise increases serum irisin levels significantly in Relapsing–Remitting Multiple Sclerosis (RRMS) patients compared to patients without intervention [37]. A study by Makiel et al. showed aerobic exercise increases irisin levels in males with metabolic syndrome (MetS) [38]. Another study reported that endurance athletes exhibit high levels of brain-derived neurotrophic factor (BDNF) and irisin in their blood, compared to sedentary controls [39], and higher scores on cognitive function in behavioral tests.

Sedentary-aged rats display lower BDNF and irisin levels in the heart, liver, and plasma compared to young and aged exercised groups [40]. Irisin levels increase in both the muscle tissue and serum of male mice after acute exercise [41]. Regular voluntary aerobic exercise in male mice results in a decrease in anxiety levels accompanied by an increase in irisin levels in various tissues, including the brain, brown adipose tissue, white adipose tissue, kidney, and pancreas [42]. Resistance training effectively elevates irisin levels in the skeletal muscles of type 2 diabetic rats, indicating its potential as an intervention for improving muscle function in diabetes [43]. Interestingly, irisin is also present in circulating extracellular vehicles (EVs), lipid-bound vesicles secreted by cells into the extracellular space [44], and sustained exercise in mice leads to the accumulation of circulating extracellular vehicles (EVs) [44].

## 4. Biological Actions of Irisin

**Figure 1:**
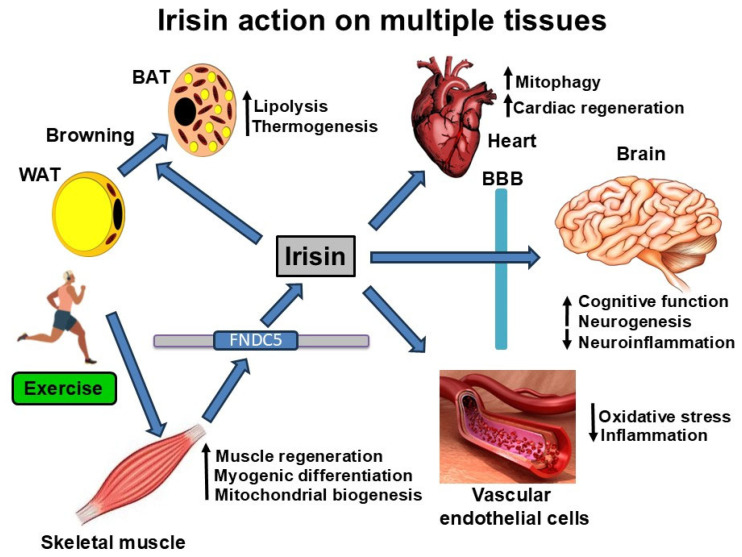
Irisin actions in multiple tissues: Irisin is cleaved and released from the trans membrane protein FNDC5 in response to exercise. It acts on multiple tissues, mediating the beneficial actions of exercise. It causes the browning of white adipose tissue (WAT) to brown adipose tissue (BAT). It passes through the blood barrier (BBB) and exerts neuroprotective actions in the brain. It increases mitophagy in the heart and mitochondrial biogenesis in the skeletal muscle. Irisin also decreases oxidative stress and inflammation in vascular endothelial cells. Irisin actions in the peripheral tissues have added benefits in aging Alzheimer’s patients with comorbidities, including obesity, diabetes, hypertension, and cardiovascular diseases.

Irisin actions on multiple tissues are summarized in Figure 1. In this section, we focus on irisin actions in the skeletal muscle and brain. Its actions in other tissues, including adipocytes, the heart, and endothelial cells, are covered under relevant disease conditions.

### 4.1. Irisin Actions in Skeletal Muscle

Irisin acts in skeletal muscle in an autocrine/paracrine fashion [45]. In mice, irisin injection induces significant muscle hypertrophy and enhances grip strength in uninjured muscle, while also improving muscle regeneration and hypertrophy following skeletal muscle injury [46]. Ectopic injection of recombinant murine irisin peptide in *mdx* mice, a model of muscular dystrophy, increases muscle weight, enhances grip strength, and reduces fibrotic tissue accumulation and myofiber necrosis [47]. Additionally, irisin treatment improves sarcolemma instability, highlighting its potential therapeutic value for muscular dystrophy [47]. Irisin promotes myogenic differentiation and myoblast fusion by upregulating pro-myogenic and exercise response genes in myotubes, primarily through IL-6 signaling activation [46]. In cultured C2C12 myotubes, irisin upregulates key genes associated with mitochondrial function and biogenesis, including PGC-1α, NRF1, TFAM, GLUT4, and UCP3 [48], and enhances mitochondrial content and oxygen consumption [49]. Irisin also translocates glucose transporter type 4 to the plasma membrane in differentiated skeletal muscle cells, leading to enhanced glucose uptake by increasing AMPK phosphorylation [50].

### 4.2. Irisin Actions in the Brain

Irisin crosses the blood–brain barrier (BBB) and stimulates the expression of BDNF, improves cognitive function, enhances neurogenesis, and modulates synaptic plasticity [51]. Lower serum irisin levels are associated with mood disturbances, particularly among a cohort of chronic obstructive pulmonary disease (COPD) patients with lower BDNF levels [52]. Additionally, irisin converts microglia from a proinflammatory M1 phenotype to an anti-inflammatory M2 phenotype [53]. A study with exercised mice showed a positive correlation between brain irisin levels and decreased anxiety as measured with an open field test and elevated plus maze test [42]. Peripheral administration of irisin in rats resulted in decreases in the levels of dopamine metabolite, a 3,4-dihydroxyphenylacetic acid in hypothalamic nuclei associated with feeding behavior [54]. Irisin treatment in C57BL/6 mice increases the mRNA levels of anorexigenic and neurotrophic genes, including CART, POMC, NPY, and BDNF, in the brain [55]. Mice subjected to physical inactivity display increases in adiposity, anxiety, depressive symptoms, and cognitive impairment [56]. These symptoms are attenuated by exercise-induced irisin, along with elevated levels of FNDC5 and PGC-1α in skeletal muscle and increases in hippocampal BDNF expression and cell proliferation. Importantly, these beneficial effects of exercise are compromised through the administration of an irisin-neutralizing antibody [56].

## 5. Irisin in the Prevention of Aging-Associated Diseases

Several clinical and preclinical studies have reported that irisin plays a key role in mediating the positive effects of exercise. The use of irisin antibodies and the deletion of the *Fndc5* gene have provided strong evidence linking exercise with irisin. This new knowledge supports the therapeutic potential of irisin in multiple disease conditions of the elderly, which is the focus of this section.

### 5.1. Exercise, Irisin, and Aging

Progressive decrease in physical activity is one of the causes of diseases in the elderly. Exercise in aging populations can be a challenge because of mobility limitations. Exercise performance has been reported to decrease with age due to lower tolerance for peripheral fatigue [57]. Aging-associated cardiovascular changes further reduce exercise capacity [58]. Aerobic capacity measured as peak VO2 has been shown to decrease with advancing age [59]. Another study reported a decrease in functional fitness for exercise due to reductions in muscle strength [60]. Thus, when elevating endogenous irisin through exercise is a challenge with advancing age, intervention with exogenous irisin can provide an alternate therapeutic approach.

Apart from exercise, certain drugs have been shown to induce the production of irisin in preclinical models. Increases in circulating irisin levels are observed after two weeks of treatment with the cholesterol-lowering drug simvastatin in healthy individuals [61]. Sitagliptin, a dipeptidyl peptidase-4 (DPP4) inhibitor that increases the endogenous GLP-1 levels, has been shown to increase serum irisin levels significantly in T2DM patients (ages 21–65 years) after 16 wks of treatment at a dose of 100 mg/day [62]. Metformin, another glucose-lowering drug, increases the levels of the irisin precursor FNDC5 and irisin in the blood of diabetic mice and in cultured myotubes [63]. Interestingly, it has been shown that hypothalamic irisin expression increases with leptin, insulin, and metformin treatments in diabetic rats [64]. Fenofibrate, a PPARα agonist, increases plasma irisin levels in obese mice [65]. All-trans retinoic acid (ATRA) administration in mice elevates circulating irisin levels along with increased *fndc5* expression in the liver and adipose tissues [66]. This study also demonstrated that ATRA treatment of murine C2C12 myocytes results in elevated fatty acid oxidation and irisin expression. Thus, a pharmacological approach that increases irisin levels in the aging population provides an alternative therapeutic strategy.

### 5.2. Exercise, Irisin, and Alzheimer’s Disease

Alzheimer’s disease (AD) is an aging-associated disease characterized by progressive memory loss and cognitive decline. The major causes of AD are the deposition of beta-amyloid plaques and phosphorylation of tau leading to neurofibrillary tangles [67]. In addition, AD pathogenesis involves neuroinflammation, microglial activation, mitochondrial dysfunction, and energy deprivation in the brain [68,69]. In this section, we will first examine the positive effects of physical exercise on cognitive function and AD pathology before discussing the role of irisin as a possible mediator. A randomized training-controlled trial with 120 older adults reports that aerobic training increases the size of hippocampus and improves memory [70]. A meta-analysis of studies with 2242 AD patients reveals a nonlinear relationship between exercise and cognitive improvement [71]. Moderate- to high-intensity aerobic exercise on a stationary bike (1 h, three times a week for 16 weeks) preserves cognitive function in patients with mild AD [72].

Several studies have suggested irisin as a mechanistic link between exercise and improved cognitive function. FNDC5/irisin levels are reduced in the hippocampus and cerebrospinal fluid in AD patients compared with cognitively intact individuals [31]. In another study, RNA sequencing data reveal a notable decrease in *Fndc5* expression, particularly in the para-hippocampal gyrus in AD patients compared to cognitively intact individuals [73]. Genetic deletion of *Fndc5*/irisin in the brain impairs long-term potentiation and novel object recognition memory in aged mice (21–24 months old), but not in young mice (8–10 weeks old) [21]. Conversely, *Fndc5*/irisin overexpression in the brain rescues synaptic plasticity and memory in AD mouse models [21]. Delivering irisin to the dentate gyrus of global *fndc5* knockout mice restores cognitive function in AD mice [21]. This study also shows that peripheral delivery of irisin by adeno-associated viral overexpression in the liver results in the reversal of cognitive deficit and AD pathology. Lourenco et al. (2019) [31], in an elegant study, provided multiple lines of evidence suggesting the neuroprotective effects of irisin in AD. First, they observed that *Fndc5*/irisin expression is reduced in the cerebrospinal fluid/hippocampi of late-stage AD patients compared to age-matched early AD or control individuals as well as in AD mice. They demonstrated that knockdown of *Fndc5*, through the intracerebroventricular infusing of lentivirus (sh*Fndc5* 1 or 2) in C57/BL6 mice, impairs the maintenance of hippocampal long-term potentiation (LTP) and object recognition memory [31]. Conversely, bilateral intrahippocampal infusion of recombinant irisin prevents Aβ oligomer-induced impairment in synaptic plasticity and the novel object recognition memory test. Peripheral overexpression of *Fndc5*/irisin, by injecting the Ad*Fndc5* vector into the caudal vein of mice, salvages memory impairment in Aβ-infused mice. Importantly, blockade of FNDC5/irisin in peripheral (intraperitoneal administration of anti-FNDC5) or in the brain (intracerebroventricular injection of lentiviruses harboring sh*Fndc5*) attenuates the neuroprotective actions of physical exercise on synaptic plasticity and memory in Aβ-infused mice [31]. These findings provide key evidence that irisin mediates the beneficial actions of exercise in the brain. However, exercise could act through other pathways to slow cognitive decline in AD. Exercise has been shown to secrete other soluble factors, including hepatokines, osteokines, and adipokines, which may also exert beneficial actions at the systemic level and indirectly improve cognitive function [74].

Although studies have shown irisin to be effective in both males and females in general, some studies have investigated the sex-dependent effects of exercise and irisin [75]. Specifically, a recent correlation study of dementia scores with amyloid deposition and CSF irisin levels reported that irisin is likely to be more beneficial in females [76]. Another study reported that irisin treatment of transgenic htau mice with irisin for one month resulted significant decreases in phosphorylated (Ser202) tau in the hippocampus of female mice but not in male mice [77]. However, more studies are needed to ascertain a clear advantage in the case of females with respect to the benefits of exercise and irisin on cognitive function.

### 5.3. Irisin and Other Neurodegenerative Diseases

Several studies have reported beneficial actions of irisin in other neurodegenerative diseases, viz., Parkinson’s disease (PD), multiple sclerosis (MS), cerebral ischemia, ischemic stroke, etc. Irisin has been shown to reduce α-synuclein pathology and protect neurons against α-synuclein preformed fibril (PFF)-induced neurotoxicity at least in part by increasing the lysosomal degradation of pathologic α-synuclein [78]. This study also showed a decrease in the loss of dopaminergic neurons and improvement in motor outcomes in the α-synuclein preformed fibril (PFF) mouse model of sporadic PD. Furthermore, irisin reduces the motor deficits induced by α-synuclein PFF, as assessed behaviorally with the pole test and grip strength test. Exercise-induced reduction in neuronal apoptosis in MPTP-induced PD mice is counteracted by the treatment of cyclo RGDyK, an inhibitor of irisin signaling [79]. It has been suggested that exogenous irisin administration can be a promising exercise-mimicking therapeutic approach for PD patients because of their inability to carry out regular exercise [80]. Multiple sclerosis (MS) is another chronic progressive neurodegenerative disease of the central nervous system [81,82]. Aerobic exercise increases irisin serum levels and has a positive effect on both cognitive and psychological functioning through improvements in depression, cognitive performance, and fatigue states in Relapsing–Remitting MS (RRMS) patients compared to RRMS patient without intervention [37]. Irisin administration is reported to exert neuroprotective effects in experimental autoimmune encephalomyelitis (EAE) mice, a model for MS through a mechanism involving the suppression of microglial activation [83]. Exercise-induced irisin enhances cognition in a mouse model of cerebral ischemia by upregulating the expression of klotho, a protein that helps in controlling insulin sensitivity, MnSOD, and FOXO3a, and reduces ROS generation [84]. Conversely, the neuroprotective effects of irisin were lost in klotho knockout mice, suggesting the role of klotho in mediating irisin action in cerebral ischemia mouse model. Furthermore, irisin has been shown to exert neuroprotective effects against ischemic injuries and possesses anxiolytic and antidepressant properties [51]. Also, irisin reduces oxidative stress and enhances blood–brain barrier integrity in newborn Sprague-Dawley rats following hypoxic–ischemic injury [85]. The protective effects of irisin against blood–brain barrier (BBB) dysfunction following focal cerebral ischemia/reperfusion in rats are accompanied by decreases in the expression and activity of matrix metalloproteinase-9 (MMP-9) in the cortex [86]. Electroacupuncture following ischemic stroke promotes motor function recovery and alleviates neuronal death through the upregulation of irisin expression in the peri-lesional cortex in rats, suggesting a protective role of irisin in ischemic stroke [87]. In another study, irisin was shown to exert beneficial effects following I/R injury in rats through the downregulation of the TLR4/MyD88/NF-κB pathway [88]. Intracerebroventricular injection of irisin in rats increases the UCP2-5 mRNA expression in different areas of the brain [89]. Irisin also reduces brain edema caused by BBB dysfunction after traumatic brain injury (TBI) in C57BL/6J mice by promoting the expression of UCP2 on the mitochondrial membrane of neurons and contributes to the neuroprotective effects [90]. Irisin administration in mice attenuates brain injury by reducing apoptosis through a mechanism involving the downregulation of Bax and caspase-3 expression and upregulation of Bcl-2 and BDNF expression in the ischemic brain cortex [91].

## 6. Irisin and Comorbidities of AD

Metabolic syndrome (MetS), a cluster of conditions including high blood pressure, low HDL cholesterol, visceral obesity, high triglycerides, and insulin resistance, together raise the risk of cardiovascular diseases, diabetes, and stroke [92]. Physical inactivity (sedentary behavior, lack of moderate/vigorous physical activity) is considered an important risk factor for developing MetS [93]. Thus, regular exercise is strongly recommended as a lifestyle change for people with metabolic syndrome [94]. We demonstrated that metabolic syndrome can accelerate AD pathogenesis [95,96,97,98]. Irisin exerts protective effects against comorbidities (obesity, diabetes, and cardiovascular disease) by promoting the browning of white adipose tissue and by reducing inflammation and insulin resistance [28,99]. Therefore, we discuss the effects of exercise and irisin on diseases that coexist with aging AD patients.

### 6.1. Irisin and Diabetes

Chronic type 2 diabetes (T2DM) is known to result in complications affecting peripheral tissues. Its effect on the CNS is increasingly being recognized. Irisin levels are significantly lower in T2DM patients compared to normal subjects [100]. Decreases in serum irisin in diabetic mice are restored following treadmill exercises (5 days/week at 7–11 m/min for 60 min/day) for 8 weeks, which also ameliorates diabetes-associated glucose intolerance and bone loss [101]. Conversely, the benefits of physical exercise are compromised after the deletion of the *Fndc5* gene in mice [102]. Decreases in serum irisin levels and *Fndc5*/irisin gene expression in the heart of T2DM rat are ameliorated by exercise [103]. A reduction in hippocampal irisin expression and elevated soluble Aβ42 levels are observed in rats following intracerebroventricular (ICV) injection of streptozotocin [104]. This study also reports that combining swimming and carnosine intake enhances the irisin levels, improves cognitive function, and decreases AD biomarkers, including soluble β-amyloid peptide and phosphorylated tau protein. Irisin administration in STZ-induced insulin-deficient diabetic mice and obese mice models, improves glycemic control and promotes weight loss [100]. Irisin increases β-cell functional mass, augments insulin biosynthesis and glucose-stimulated insulin secretion (GSIS) [100]. Interestingly, insulin decreases serum irisin levels in normal rats, whereas irisin levels increase after insulin treatment in diabetic rats [64]. Irisin signaling plays a role in exercise-linked beneficial effects on myocardial injury and excessive mitochondrial fission in diabetes rats by a mechanism involving elevated AMPK phosphorylation [103]. Irisin has been shown to improve insulin resistance by increasing oxidative phosphorylation and mitochondrial content in C2C12 myoblast cells through the p38/MAPK-PGC1-α axis [49].

### 6.2. Irisin and Obesity

In populations with obesity, irisin levels are positively associated with muscle mass and resting energy expenditure but negatively associated with body fat percentage, insulin resistance, and triglyceride levels [105]. Irisin levels are lower in obese animal models, and its administration improves glycemic control and promotes weight loss [100]. Exercise increases the circulating levels of irisin in subjects with overweight or obesity [106]. Swimming exercise-induced elevation of irisin contributes to the reduction in body fat mass in high-fat-fed obese rats [107]. Rats subjected to exercise on a motorized treadmill display elevated irisin levels [108]. These circulating irisin levels are inversely associated with abdominal visceral and epididymal fat and positively correlated with indices of cardiac function. Another study on male Sprague-Dawley obese rats (8 weeks of age) showed that high-intensity interval training (HIIT) exercise is more effective in inducing irisin uptake from circulation into adipose tissue and in maintaining the irisin levels in skeletal muscle [109]. Lentivirus-mediated *Fndc5* overexpression in high-fat-fat obese mice leads to increased energy expenditure and insulin sensitivity [110]. Adipose tissue browning is a physiological process that increases energy expenditure, improves glucose homeostasis, and combats against obesity [111]. Swimming exercise intervention in high-fat-fed obese rats upregulates irisin and induces the browning of white adipocytes, alleviating the development of obesity [112]. Similarly, irisin intervention in HFD-induced obesity in mice induces white adipose tissue browning, improving blood glucose levels, insulin resistance, lipid metabolism, energy expenditure, and UCP1 expression [113]. Thus, irisin’s multifaceted effects on adipocyte phenotype and metabolism suggest that it is a promising target for obesity prevention [114].

### 6.3. Irisin and Cardiovascular Diseases (CVDs)

Physical inactivity, diet, and smoking are among the contributing factors for the cause of CVDs. In a cross-sectional study, serum irisin levels were found to be lower in patients with congestive heart failure (CHF) [115]. Chronic administration of irisin through osmotic pumps or single bolus injections in Sprague-Dawley rats increases the expression of FNCD5, Raf1, CPT1, IGF-1, and CALCIN and decreases PGC1α, Nox4, and Mfn1 expressions, suggesting a cardioprotective role for irisin [116]. Resistance exercise has been shown to enhance cardiac function by regulating mitophagy through the irisin/FNDC5-PINK1/Parkin-LC3/P62 pathway in a myocardial infarction model in mice [117]. A study by Zhao et al. shows that cardiac progenitor cells (CPCs), treated with irisin before transplantation into the infarcted myocardium in mice, promote cardiac regeneration and neovascularization [118]. FNDC5 deficiency in mice aggravates vascular stiffness, senescence, oxidative stress, inflammation, and endothelial dysfunction in 24-month-old naturally aged and Ang II-treated mice [119]. *Fndc5* knockout mice abrogate exercise-induced protection against Ang II-induced vascular stiffness and senescence [119]. Irisin also improves endothelial junctions and endothelial barrier integrity in LPS-treated endothelial cells (HMVECs and HUVECs) by binding to integrin αVβ5 receptor and through suppression of the Src-MLCK-β-catenin pathway and activation of the AMPK-Cdc42/Rac1 pathway [120].

## 7. Mechanism of Irisin Action

Irisin actions are mediated by its binding to integrin αV/β5 receptors followed by the stimulation of FAK/Akt/CREB pathway in osteocytes [24]. The treatment of these cells with inhibitors of integrin αV effectively prevented irisin signaling. Another study demonstrated that heat shock protein 90α (Hsp90α) facilitates the binding of irisin to integrin αV/β5 receptors [121]. Interestingly, this study also observed the increase in Hsp90α levels in muscles following exercise. Similarly, irisin/receptor binding has been demonstrated in multiple other cell types. Irisin binds to αVβ1/β5 integrins and mediates the activation of integrin-FAK pathways, in adipocyte progenitor cells (APCs) [122]. The binding of irisin to integrin αV/β5 receptors has been reported in the 3D cultures of astrocytes followed by the downregulation of ERK1/2/STAT3 pathways and the induction and release of the neprilysin, an enzyme involved in Aβ degradation [123]. Further characterization showed the irisin–integrin interaction to be unconventional because irisin lacks RGD motif present in integrin ligands and the glycosylation of irisin enhances its affinity for integrin [121]. The irisin/αV/β5/AMPK signaling pathway is involved in decreasing neuroinflammation and neuronal apoptosis after intracerebral hemorrhage (ICH) in mice, suggesting that irisin activates AMPK to regulate energy metabolism [124]. Administration of 5-aminoimidazole-4-carboxamide riboside (AICAR), an agonist of AMPK has been shown to improve cognition and hippocampal neurogenesis in mice [125,126]. AICAR is also known to induce metabolic genes in sedentary mice [127]. Irisin also acts through the cAMP/PKA pathway, as reported by some studies. For example, irisin increases basal lipolysis in cultured 3T3-L1 adipocytes through a mechanism involving the cAMP-PKA-HSL/perilipin pathway [110]. Irisin stimulates the cAMP-PKA-CREB pathway and induces BDNF expression in human cortical slices and in mouse hippocampal slices [31]. Adenoviral-mediated irisin expression in hippocampal cultures elevates BDNF levels along with ERK1/2 activation and reduction in Aβ oligomer-induced oxidative stress [128]. Another study has shown that irisin upregulates the expression of uncoupling protein-1 (UCP-1; a regulator of thermogenic capability of brown fat), and this effect is mediated by the p38 MAPK and ERK1/2 signaling pathways [129]. These effects of irisin on UCP-1 are lost in the presence of the p38 MAPK inhibitor (SB203580) and ERK1/2 inhibitor (U0126) in primary rat adipocytes and 3T3-L1-derived adipocytes [129]. Thus, irisin-mediated signaling appears to vary in a cell-type-dependent manner.

### Irisin and Sirtuins

Sirtuins, a family of seven proteins, are nicotinamide adenine dinucleotide (NAD)-dependent protein deacetylases that play key roles in metabolism, longevity, and inflammation [130]. Among sirtuins, SIRT1 and SIRT3 have been studied extensively for their roles in the deacetylation/activation of nuclear and mitochondrial proteins, respectively [131]. Irisin and sirtuins share the common mechanism of being induced by exercise, suggesting potential crosstalk between these two pathways [102]. For example, muscle biopsy of male cyclists after 3 days of exhaustive training shows an increase in PGC-1α and *Sirt1* mRNA expression [132]. SIRT1 activity increases in the heart and adipose tissue after 6 weeks of a treadmill training program in aged rats [133]. A clinical study in vitamin D-deficient type 2 diabetic patients showed that following supplementation with vitamin D, glycemic control was accompanied with increases in the plasma levels of irisin and SIRT1 [134]. The expressions of both irisin and SIRT1 are increased in the soleus muscle tissue of diabetic rats trained by running on a treadmill for 12 weeks [135]. The AMPK/SIRT1/PGC-1α pathway is activated in the hippocampus of rats undergoing swimming training [136]. Irisin action is also linked to SIRT3, another sirtuin and a key player in mitochondrial metabolism. For example, endurance training for 8 weeks increases SIRT3 protein levels in the brain, liver, and skeletal muscles of people aged 18–30 years and over 65 years [102,137]. Moreover, FNDC5 deficiency-induced disruption of mitochondrial bioenergetics after TBI in mice is ameliorated by SIRT3 activation [138]. FNDC5 deficiency impairs SIRT3 expression by enhancing ubiquitin degradation of transcription factor nuclear factor erythroid 2-related factor 2 (NRF2) after TBI in mice [138]. Moreover, *fndc5* gene silencing in HT22 cells, a mouse hippocampal cell line, leads to the downregulation of SIRT3 at both transcriptional and protein levels [138]. The neuroprotective effects of exercise in a PD mouse model involves the irisin/AMPK/SIRT1 signaling pathway [79]. Irisin alleviates hepatic steatosis in a mouse model by improving mitochondrial fusion through the PKA/SIRT3/mTOR pathway [139]. In neuron-specific *Sirt3* knockout mice, FNDC5 fails to rescue TBI-induced mitochondrial damage and brain injuries, suggesting FNDC5/irisin plays a protective role against acute brain injury by promoting SIRT3-dependent mitochondrial biogenesis [138]. Irisin has been shown to protect against cerebral ischemia–reperfusion injury by restoring SIRT3 expression [140]. This study also demonstrated that the neuroprotective effects of irisin in cultured PC12 cells, a neuronal cell line, were lost following *Sirt3* silencing with siRNA and through treatment with 3-TYP, an SIRT3 inhibitor. Our lab characterized the role of SIRT3 in brain mitochondrial metabolism [98]. We reported that deletion of the Sirt3 gene in Alzheimer’s transgenic mice results in the exacerbation of amyloid pathology and neuroinflammation [96]. Altogether, exercise, irisin, and SIRT3 hold promise to improve the cognitive decline in neurodegenerative diseases, including AD.

## 8. Therapeutic Potential of Irisin

Exercise as a lifestyle modification is generally suggested in the management of aging-associated diseases. However, this approach may be difficult with some aged individuals, especially those with chronic diseases. Decreased muscle strength and cardiovascular changes are some of the causes. In such cases, pharmacological interventions that mimic the benefits of exercise can be considered. Several studies have used irisin antibodies and the deletion of the *Fndc5* gene to suggest that irisin plays a key role in mediating the positive effects of exercise. Therefore, the therapeutic potential of irisin in aging-associated diseases is increasingly being recognized.

The beneficial actions of exogenous irisin have been demonstrated in multiple disease models. Because irisin can cross the blood–brain barrier and exert neuroprotective actions in the brain, its actions in neurodegenerative diseases have been tested in many clinical and preclinical studies with encouraging results, especially in the case of AD. Recent studies have reported that AD often coexists with other diseases, including obesity, diabetes, hypertension, and cardiovascular diseases. Interestingly, irisin exerts protective effects against these comorbidities as well through its pleiotropic actions, including the browning of white adipose tissue and decrease in insulin resistance and inflammation.

In addition to the administration of irisin, drugs that are known increase endogenous irisin production also hold promise. Cholesterol-lowering drug simvastatin and anti-diabetic drugs metformin and sitagliptin have been reported to elevate irisin production in the body. Studies have also explored the potential of oral agents that can activate the components of the signaling pathways stimulated by irisin. Some of the components of these pathways that have been targeted include AMPK and PPARδ. Thus, investigation of irisin biology can lead to multiple therapeutic approaches.

## Data Availability

No new data were created or analyzed in this study. Data sharing is not applicable to this article.

## Abbreviations

AD, Alzheimer’s disease; ADHF, Acutely Decompensated Heart Failure; ADSCs, Adipose tissue-derived Mesenchymal Stromal Cells; AE, aerobic exercise; AHF, acute heart failure; AMPK, AMP-activated protein kinase; ATRA, all-trans retinoic acid; BBB, blood–brain barrier; BDNF, brain-derived neurotrophic factor; CPCs, cardiac progenitor cells; EA, electroacupuncture; ERP, event-related potential; EVs, extracellular vesicles; FGF21, fibroblast growth factor 21; FNDC5, fibronectin type III domain-containing protein 5; GSIS, glucose-stimulated insulin secretion; HF, heart failure; HFD, high fat diet; HIIT, high-intensity interval training; ICV-STZ, intracerebroventricular injection of streptozotocin; LVEDP, left ventricular end-diastolic pressure; MDD; major depressive disorder; MetS, metabolic syndrome; MMP-9, matrix metalloproteinase-9; MAPK, mitogen-activated protein kinase; MICE, moderate-intensity continuous exercise; MICT, moderate-intensity continuous training; MI/R, myocardial ischemia/reperfusion injury; MS, multiple sclerosis; NAFLD, non-alcoholic fatty liver disease; PD, Parkinson’s disease; PGC-1α, peroxisome proliferator-activated receptor gammacoactivator-1 alpha; PFF, preformed fibril; RRMS, Relapsing–Remitting MS; RPP, rate pressure product; RT, reaction time; S1P, sphingosine-1-phosphate; TBI, traumatic brain injury; T2DM, type 2 diabetes mellitus; UCP1, uncoupling protein 1; VAT, visceral adipose tissue; WAT, white adipose tissue.
